# Small G Protein StRab5b positively regulates potato resistance to *Phytophthora infestans*

**DOI:** 10.3389/fpls.2022.1065627

**Published:** 2023-01-09

**Authors:** Zaimin Tian, Zhiwei Zhang, Liru Kang, Min Li, Jian Zhang, Yan Feng, Jiang Yin, Xuechen Gong, Jun Zhao

**Affiliations:** ^1^ College of Horticulture and Plant Protection, Inner Mongolia Agricultural University, Hohhot, China; ^2^ College of Agriculture and Forestry Science and Technology, Hebei North University, Zhangjiakou, China; ^3^ Inner Mongolia Academy of Agricultural and Animal Husbandry Sciences, Hohhot, China

**Keywords:** Small G proteins, potato late blight, antioxidant enzyme activities, plant disease resistance, gene expression, hormone signaling pathways

## Abstract

Rabproteins are the largest members of the small G protein family and are widely distributed in eukaryotes. It comprises eight subfamilies and is responsible for regulating vesicle transport, plant growth and development, and biotic and abiotic stress responses. In this study, the small G protein gene *StRab5b* was cloned from potato, and its biological information, expression profile and induced expression level, overexpression and gene silencing were examined on regulating potato resistance to *Phytophthora infestans* using PCR, qPCR and Virus-induced gene silencing (VIGS). Our results indicate that the amino acid of StRab5b shows the highest and lowest homology with NbRab5b in *N. benthamiana* and StRab in potato respectively. *StRab5b* expression varied among different potato tissues and varieties, and was induced by *P. infestans* infection. Transiently ectopic expression of *StRab5b* in *N. benthamiana* enhanced its resistance to *P. infestans*, whereas, silencing of *StRab5b* and its homologous gene facilitated pathogen infection in potato and *N. benthamiana* respectively. Furthermore, stable expression of the *StRab5b* gene in potatoes enhanced its redox-stress response capacity, as manifested by the accumulation of H_2_O_2_ in infected leaves and subsequent increase in the activity and expression of ROS scavenging enzymes, thereby attenuating the development of *P. infestans* and ultimately reducing the lesions on infected potato leaves. In addition, the *LOX* gene transcripts and JA level were upregulated rapidly after inoculation with *P. infestans*. Collectively, our results suggest that *StRab5b* positively regulates the resistance against potato late blight (PLB) *via* JA-mediated defense signaling pathway.

## 1 Introduction

The potato (*Solanum tuberosum* L.) is the most important non-gramineous crop worldwide. However, its tuber yield and quality are devastatingly affected by PLB caused by *P. infestans*. In China, in the early 1950s and the 1960s, PLB caused yield losses amounting to approximately 1 billion dollars (Li et al., 1997). Furthermore, the frequent variation in dominant races of *P. infestans* in potatoes in recent years has been considerable, resulting in a loss of disease resistance (Saville et al., 2016). At present, it is extremely difficult to control PLB, and an effective way is urgently required to excavate novel functional genes for investigating their resistance functions.

Plants have developed sophisticated pathogen-surveillance systems and can mount defense responses against microbial attack. Microbial parasites in the environment constantly challenge plants, which in turn, defend themselves through the innate immune system. Specifically, plants detect potential invaders, including bacteria, fungi, and oomycetes, mainly through a two-layered immunity system. First, when the host plants defend pathogen infection, the pattern recognition receptors (PRRs) located on the cell membrane recognize the pathogen associated molecular patterns (PAMPs) of *Phytophthora* pathogens, and elicit the first layer of immune system (PAMP-triggered immunity, PTI) (Naveed et al., 2020). Microbe-associated molecular patterns (MAMPs) are motifs of microbes which can be recognized by plants in response to infection (Robinson and Bostock, 2015). Then, MAMPs triggered PTI defense responses that can effectively counteract potential microbes. Second, pathogens secrete and deliver effectors into the host cells, that neutralize the functions of immune regulators (Saville et al., 2016). Resistance proteins (R) recognize pathogen effectors, activating a rapid immune response known as effector-triggered immunity (ETI), which results in host cell death, a phenomenon known as the hypersensitive response (HR). These pathogen effectors can evolve new functions through mutations to suppress immunity and promote disease progression (Wang et al., 2019).

During the establishment of HR, Salicylic acid (SA), JA, and ethylene (ET) biosynthesis are activated through PTI, ETI, and systemic acquired resistance (SAR). The accumulation of SA, JA and ET leads to enhanced resistance to pathogens in plants (Li et al., 2004). Particularly, recent studies have reported that the transcription factor *StbZIP61* is involved in the SA signaling pathway that regulates the defense response of potato against *P. infestans* (Zhou et al., 2018) and enhances potato resistance to salt stress (Efimova et al., 2019). Similarly, activating JA and ET signaling, instead of SA signaling by eliciting β-cryptogein, triggers resistance to *P. infestans* (Starý et al., 2019).

In addition, when plants are subjected to biotic or abiotic stress, rapid accumulation of reactive oxygen species (ROS) occurs, leading to programmed cell death (PCD) and HR. However, cells have evolved a set of ROS-scavenging enzymes, including superoxide dismutase (SOD), peroxidase (POD), catalase (CAT), and ascorbate peroxidase (APX) to balance cellular ROS levels (Beers and Sizer, 1952). Recent evidence suggests that the higher activities of POD and SOD play key roles in scavenging excessive ROS in late plant-pathogen interaction. Higher levels of H_2_O_2_ were also represented in potato transgenic plants after inoculation with *P. infestans* (Zhang et al., 2014). Zhang et al. (2020) found that ROS can be scavenged by SOD, POD, and CAT activities. In sweet potato, SOD and APX activities of transgenic plants were increased under salt stress (Yan et al., 2016). In conclusion, ROS and antioxidant enzymes are involved in the signal pathway of plant-pathogen interaction and stress response.

The RAS superfamily of small G proteins are divided into five subfamilies: RHO, RAB, RAS, RAN and ARF. RAB proteins belong to the largest subfamily of RAS superfamily. RAB GTPase generally regulates its activity through two regulatory proteins: Guanine nucleotide exchange factors (GEFs) are used to dissociate the GDP to form an active GTP-RAB-effector, and GTPase activating proteins (GAPs) are used to decompose GTP into GDP. GDP-RAB combines with GDI to form inactive GDP-RAB-GDI, thus entering the next regulation cycle (Ebine and Ueda, 2009). RAB proteins are widely distributed in plants, animals, and microorganisms. To date, 52 *Rab* genes have been cloned from rice (*Oryza sativa* L.) and 57 from *Arabidopsis thaliana* (Rutherford and Moore, 2002; Du et al., 2013). In potato, the *Rab* gene *StRab5b* is located on the 11^th^ chromosome of the genome (https://solgenomics.net/locus/82544/view). Previous studies have shown that Rab proteins regulate both abiotic and biotic stress responses in plants, plant hormone-mediated signal transduction, plant growth and development, and vesicular trafficking (Yamaguchi-Shinozaki et al., 1990; Moshkov and Novikova, 2008; Szumlanski and Nielsen, 2009; Falchi et al., 2010; Gao et al., 2012). Research of plant bacterial diseases showed that the overexpression of *OsRab11* in rice resulted in elevated resistance to the pathogen *Pseudomonas syringae* through induced JA response genes (Hong et al., 2013). Small G protein, *VmRab7*, regulates the vegetative growth and pathogenicity of *Valsa mali*, and knockout of *VmRab7* blocks autophagy (Zhang et al., 2021). In previous studies on plant fungal diseases, *Rab2* was significantly expressed in the early stage of infection by wheat leaf rust (*Puccinia triticina*). The active *OsRac1* caused HR responses and substantially reduced disease lesions against rice blast fungus (Ono et al., 2001). In Arabidopsis, *RabG3bOX*-transgenic plants displayed unrestricted hypersensitive PCD against a fungal toxin and a fungal pathogen *Alternaria brassicicola* (Kwon et al., 2009). Additionally, higher expression of *TaRab7* implied that it was related with defense to wheat-stripe rust fungus (Liu et al., 2012). Moreover, the overexpression of the small G protein *StRab* caused small lesions on potato leaves after inoculation with *P. infestans* (Gao et al., 2012), indicating that the *Rab* genes and their associated proteins show potential to influence disease progression. However, limited research has been done on the resistance of small G protein to PLB.

In this study, we aimed to elucidate the role of the small G protein *StRab5b* in the regulation of potato resistance against late blight. For this purpose, the *StRab5b* gene was cloned, gene sequences and expression profile were analyzed; transient expression and gene silencing were performed in *N. benthamiana* and potato. Stable expression of *StRab5b* in potatoes was obtained and used to confirm its role in regulating potato resistance against *P. infestans*. These results lay a stone for resistant breeding against potato late blight.

## 2 Materials and methods

### 2.1 Plant material, *P. infestans* strain, and primers

Potato cultivar ‘Desiree’ was used for gene amplification and genetic transformation. Plants were grown *in vitro* on Murashige and Skoog (MS) medium, under a luminous flux intensity of 3,000–4,000 Lx, 80% relative humidity, and a photoperiod of 16 h/8 h light/dark, at 25°C. The *P. infestans* strain (14-3) was kindly provided by Prof. Francine Gover, Wageningen University and Research (WUR), Netherlands. Tubers of six potato varieties ‘Zihuabai’ (ZHB), ‘Desiree’ (DXR), ‘Longshu No.7’ (L7), ‘Zhongshu No.4’ (ZS4), ‘Atlantic’ (DXY) and ‘Shepody’ (XPD), were planted in a nutrient pot and cultivated in a chamber under a luminous flux density of 18,000 Lx, 75% relative humidity, and a photoperiod of 16 h/8 h light/dark, at 25°C. These plants were used for expression profile analysis. Desiree seedlings cultivated in pots for 20 d were selected for induced expression.

Primers were designed using Primer 5.0 software and synthesized by the Beijing Hooseen Biotechnology Company, China. The primer sequences used in this study are listed in **Supplementary Table 1**.

### 2.2 *P. infestans* culture and zoospore suspension preparation

Luria-Bertani liquid medium (10 g tryptone, 5 g yeast extract, and 5 g NaCl) was prepared, and H_2_O was added to bring the volume to 1,000 mL. YEB liquid medium (5 g beef extracts, 5 g tryptone, 5 g sucrose, 1 g yeast extract, MgSO_4_·H_2_O 0.5 g) with pH 7.0 was prepared, and H_2_O was added to bring the volume to 1,000 mL. Agar powder (15 g) was added to each mixture media to prepare the corresponding solid medium. *P. infestans* was induced to produce zoospores at 4°C for 4 h. Zoospore suspensions were adjusted to a concentration of 5 × 10^5^ mL^-1^ sporangia using a hemocytometer (Neubauer-improved, Germany). Approximately 10−15 μL of spore suspension was inoculated on plant leaves.

### 2.3 RT-PCR and RT-qPCR analysis

Relative expression quantitation was performed using Quantity one software for semi-quantitative RT-PCR (*St Rab5b* and *PDS*), and *GAPDH* was used as a reference gene. Q-PCR was performed on a LightCycler (96, Roche, Basel, Switzerland) using the SYBR Premix Ex Taq II Kit (TaKaRa Bio, Shiga, Japan). We analyzed the expression levels of *ACS*, *LOX*, *NPR1*, *SOD*, *APX1*, *POD*, *CAT2*, and *PIO* and used *Actin* as a reference gene.

### 2.4 Cloning of *StRab5b* and overexpression vector construction

Total RNA was extracted from potato plantlets grown for 30 d using the RNAiso reagent (TaKaRa Bio), and genomic DNA was extracted using the DNAiso reagent (TaKaRa). The quality of RNA and DNA was determined by performing gel electrophoresis on 1% agarose gels. The first cDNA chain was synthesized according to manufacturer instructions of a HiScript II reverse transcription Kit (Nanjing Vazyme Biotechnology Co., Ltd).

The PCR-amplification mixture (50 μL) included 10 μL of 5X Prime Star Buffer (Mg^2+^ plus), 4 μL of dNTP Mix (2.5 mM), 1 μL of Primer *Rab5bF/Rab5bR*, 1 μL of cDNA template, 0.5 μL of Prime STAR HS DNA Polymerase, and 32.5 μL of ddH_2_O. Target fragments were purified using the EasyPure Quick Gel Extraction Kit (Beijing TransGen Biotech Company, China) according to manufacturer’s instructions. Plasmid extraction was performed according to the manufacturer’s instructions of the TIANprep Mini Plasmid Kit II (Beijing TIANGEN Biotech Company). PLG-Rop and pBIA1300-221 were provided by Prof. Yan Zhao, from the Institute of Genetics and Development, Chinese Academy of Sciences, Beijing, China. WUR kindly provided the *P. infestans* strains, pTRV1 and pTRV2 vectors. Fastdigest endonucleases *Kpn*I, *Bgl*II, *Xba*I, and *Sac*I were purchased from Thermo Fisher Scientific, (Waltham, MA, USA).

Phylogenetic analyses were performed with DNAMAN software. The *Rab* or *Rab5b* sequences of *Oryza sativa* (AF323991), *Nicotiana benthamiana* (DQ335217), *Solanum tuberosum* (ABK96799 and AK323357), *Hevea brasiliensis* (KC577146), *Physcomitrella patens* (AB379973), *Phoenix dactylifera* (AK287492), *Nicotiana sylvestris* (X63875), *Mangifera indica* (KF768563), *Athaliana* (D89824), *Selaginella moellendorffii* (KF516567), *Medicago sativa* (X79278), *Triticum aestivum* (X59133), *Lotus japonicus* (Z73939), and *Medicago truncatula* (BT147767) were retrieved from Genbank separately.

The *StRab5b* gene was linked to the pEASY-Blunt Simple cloning vector and transferred into DH5α cells (TransGen Biotech Company, Beijing, China). Positive DH5α colonies were screened and sequenced. Target gene purification was performed using the EasyPure Quick Gel Extraction Kit (TransGen Biotech Company). Recombinant vectors PLG-Rop and pEASY-Blunt Simple-*StRab5b* were digested with *Bgl*II and *Kpn*I to obtain the PLG-*StRab5b* vector. Recombinant plasmid PLG-*StRab5b* and vector pBIA1300-221 were digested with *Xba*I and *Sac*I to obtain the recombinant plasmid pBIA1300-*StRab5b.* Plasmid pBIA1300-*StRab5b* was transferred into *Agrobacterium tumefaciens* GV3101 for preservation.

### 2.5 Measurement of lesions on inoculated potato and *N. benthamiana* leaves

The length and width of the leaf lesions formed on the plant leaves were measured using an electronic caliper (DL91150, China). Each lesion was measured three times (Vleeshouwers et al., 1999). The lesion area (A1) was calculated using the formula: *A1 = 1/4π × length × width*.

### 2.6 Expression profile and induced expression of *StRab5b*

Inoculation method of *P. infestans* for analysis of the expression profile of *StRab5b* was described as follows. The seedlings of different potato varieties were cultured in a pot (10 cm height * 10 diameter) for 50 d. The abaxial surface of potato leaves was scratched with sterile toothpicks and inoculated with 10 uL zoospore suspension (5 × 10^5^ mL^-1^) of *P. infestans* on one side; the other side was inoculated with water as control. The inoculated leaves were placed in a plastic box with wet filter paper on the bottom and kept in an incubator (day and night 16 h/8 h, 25/18°C).

Induction of *StRab5b* after inoculation with *P. infestans*: Potato seedlings (‘Desiree’) were planted in a pot (10 cm height * 10 diameter) for 20 d, then inoculated with 10 μL of sporulation of *P. infestans* (5 × 10^5^ mL^-1^). Then, inoculated leaves were collected at 0, 12, 24, 48, 72, 96, and 108 h post-inoculation (hpi). Using potato *GAPDH* as a reference gene, RT-PCR was performed to detect the relative expression levels of *StRab5b*, and the results were analyzed using Quantity one software.

### 2.7 Transient expression of *N. benthamiana* and VIGS-mediated gene silencing in *N. benthamiana* and potato

*A. tumefaciens* containing pBIA1300-*StRab5b* and pBIA1300 were inoculated in 10 mL YEB liquid medium (100 μg/mL rifampicin and 50 μg/mL kanamycin). The oscillating culture was incubated overnight at 180 rpm, followed by centrifugation for 2 min at 7,104×*g* to collect the bacteria; then, the culture was resuspended in a liquid mixture containing 150 μM acetyleugenone, 10 mM MgCl_2_, and 10 mM MES. The bacterial solution was suitable for use when the OD_600_ value reached 0.5–0.6. The bacterial liquid (15–20 μL) was drawn using a needle-free syringe, injected into the right side of the abaxial surface of the *N. benthamiana* leaf, and incubated for 24 h (night and day alternate 16 h/8 h) at 21°C. The *N. benthamiana* leaves were inoculated with *P. infestans* spores and incubated for 4 h at 4°C before being transferred to a 21-23°C incubator with 80% humidity. The lesion area was measured at different inoculation time points.

Recombinant vectors pTRV2 and pEASY-Blunt Simple-*StRab5b* were digested with *Xba*I and *Sac*I to construct the silence-expression vector pTRV2-*Rab5b* (98% amino acid identity with *Rab5b* of *N. benthamiana*). The *Agrobacterium tumefaciens* vector pTRV1 was provided by WUR. Plasmid pTRV2-*Rab5b* was then transferred to *A. tumefaciens* GV3101. *A. tumefaciens* plasmids were resuspended with VIGS mixture containing 20 mM MgCl_2_, 100 mM MES, and 20 mM acetosyringone. The photobleaching phenotype was obtained by inhibiting the expression of endogenous phytoene desaturase gene (*PDS*). A 536 bp fragment of *StPDS* (95% amino acid identity with *PDS* of *N. benthamiana*) was linked with pTRV2 and transformed into *A. tumefaciens* GV3101 (pTRV2-*PDS*). Thereafter, *A. tumefaciens* containing pTRV1 was mixed with pTRV2-*PDS* or pTRV2-*StRab5b* at a 1:1 ratio after the OD_600_ value reached 0.5−0.6. A needle-free syringe was used to draw 20 μL of the bacterial suspension to inject into *N. benthamiana* and potato leaves. Three to four leaves of each plant were inoculated. After photobleaching, leaf samples were collected to identify the relative expression levels of *PDS*, *StRab5b* and *StRab5b* homologous gene using RT-PCR, after 25 d (*N. benthamiana*) and 60 d (potato). *GAPDH* was used as a reference.

### 2.8 Generation of transgenic potato lines

Potato stems (2 cm long) were placed in the suspended *Agrobacterium* solution, soaked for 10 min, and dried with sterile filter paper. Stems were then transferred to the co-culture medium (MS + 1.0 mg/L IAA + 0.2 mg/L GA3 + 2.0 mg/L ZT + 0.5 mg/L 6-BA, pH 5.8) and cultured in darkness for 2–3 d. Thereafter, they were transferred to the bud inductive-differentiation medium (MS + 1.0 mg/L IAA + 0.2 mg/L GA3 + 2.0 mg/L ZT + 0.5 mg/L 6-BA + 50 mg/L Kan + 300 mg/L TMT, pH 5.8), and incubated at 25°C under a light intensity of 4,000 μmol/m^2^·s, and a photoperiod of 16 h/8 h. The medium was replaced at 2-week intervals. When adventitious buds grew to 2.0 cm, they were transferred to the rooting medium (MS + 50 mg/L Kan, pH 5.8). When plantlets were formed, total leaf RNA was extracted, and positive transgenic plants were identified using PCR and q-PCR separately.

### 2.9 Trypan blue staining to monitor dead cells

Leaves infected with *P. infestans* were decolorized in anhydrous ethanol by boiling for 2 to 2.5 h. Decolorized leaves were placed in 0.5% trypan blue solution (0.5 g trypan blue dissolved in 100 mL distilled H_2_O), stained for 10–15 min, washed twice with distilled water, and then treated with lactophenol water (lactic acid, phenol, and dd H_2_O mixed in a ratio of 1:1:1).

### 2.10 DAB staining to quantify the accumulation of H_2_O_2_

Leaves infected with *P. infestans* were soaked overnight in 1 mg/mL 3,3′-Diaminobenzidine (DAB) dyeing solution (0.1 g DAB dissolved in 100 mL distilled water, pH 3.8). The leaves were decolorized by boiling for 2 h in absolute ethanol, then treated with lactophenol water (lactic acid, phenol, and water were mixed in a ratio of 1:1:1), and photographed. The H_2_O_2_ content of *P. infestans*-inoculated transgenic plants was determined as described by Zhang et al. (2014).

### 2.11 ROS scavenger enzyme activity measurement

Activities of APX CAT, POD, and SOD enzymes were determined according to manufacturer’s instructions using the corresponding kits (APX-1-W, CAT-1-W, POD-1-Y and SOD-1-W), purchased from Suzhou Keming Biotechnology Co., Ltd., (Suzhou, China). The infected leaf samples were collected from all treated plants. The data were analyzed using SPSS 16.0.

### 2.12 Determination of JA content

0.1 g smples collected from potato leaves were cut into pieces, and grinded into powders in liquid nitrogen. The content of JA in potato was determined by plant JA ELISA Kit (Jiangsu Jingmei Biological Technology Co., Ltd) (Yi et al., 2020).

## 3 Results

### 3.1 Amplification of *StRab5b*

**Figure 1:**
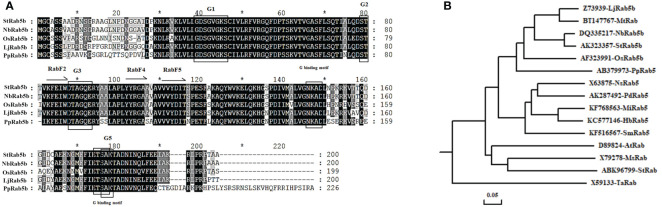
Conservative domain and phylogenetic tree analysis of StRab5b **(A)** The conserved domains of *StRab5b*. **(B)** Phylogenetic tree of *StRab5b*. The phylogenetic tree was constructed with DNAMAN v. 6.0. The accession number of each protein is as follows: *Oryza sativa* (AF323991), *Nicotiana benthamiana* (DQ335217), *Solanum tuberosum* (ABK96799 and AK323357), *Hevea brasiliensis* (KC577146), *Physcomitrella patens* (AB379973), *Phoenix dactylifera* (AK287492), *Nicotiana sylvestris* (X63875), *Mangifera indica* (KF768563), *Arabidopsis thaliana* (D89824), *Selaginella moellendorffii* (KF516567), *Medicago sativa* (X79278), *Triticum aestivum* (X59133), *Lotus japonicus* (Z73939), and *Medicago truncatula* (BT147767). The symbol * represents the middle value between two sequence numbers.

The potato *StRab5b* gene was amplified using Prime STAR HS DNA Polymerase (Takara). The fragment identified *via* agarose gel electrophoresis and the amplicon size was approximately 607 bp. To analyze the conserved domains of StRab5b, Rab 5b proteins isolated from rice, *N. benthamiana*, lotus, and *Physcomitrella patens* were retrieved from Genbank and used for multi-sequence analysis. The results suggested that StRab5b contained the basic general characteristics of Rab proteins, including RabF2 (83−87), RabF4 (YYRGA, 102−106) and RabF5 (131−135) domains, which are the conserved domains of the Rab proteins. Additionally, it also contained the GTP/GDP binding domain, phosphate binding loop G1 (39−46), G2 (79−80), and the coordination of GTP β and γ phosphate motifs G3 (88−91) and G5 (176−178), binding motifs NKAD (146−149) and ETSA (174−177) (**Figure 1A**). Therefore, the potato StRab5b protein belongs to Rab protein family.

Furthermore, the phylogenetic tree was constructed with StRab5b and the other 15 Rab proteins, derived from Oryza sativa, Nicotiana benthamiana, Solanum tuberosum, Hevea brasiliensis, Physcomitrella patens, Phoenix dactylifera, Nicotiana sylvestris, Mangifera indica, Arabidopsis thaliana, Selaginella moellendorffii, Medicago sativa, Triticum aestivum, Lotus japonicus, and Medicago truncatula, showed that StRab5b exhibited the highest similarity (95%) with NbRab5b, whereas, it showed the lowest similarity (44%) with TaRab (**Figure 1B**).

### 3.2 Expression profile analysis of *StRab5b* gene and its induced expression in potato

**Figure 2:**
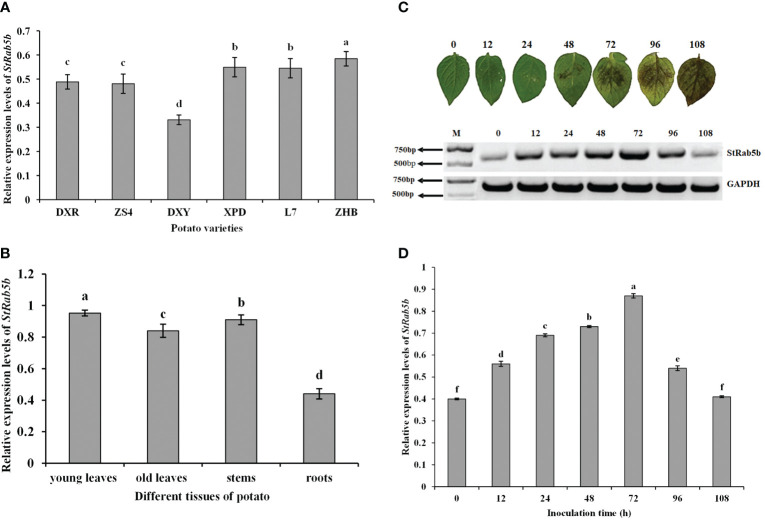
Expression pattern and inducible expression of the *StRab5b* gene in potato **(A)** Relative expression levels of *StRab5b* in six different potato varieties. **(B)** Relative expression levels of *StRab5b* in different tissues in cultivar ‘Desiree’. **(C)** Phenotype of potato leaves inoculated with *Phytophthora infestans* and the relative expression levels of *StRab5b* in cultivar ‘Desiree’ at 0–108 hpi. Results were obtained from three biological replicates. **(D)** Quantification of relative expression of *StRab5b* in cultivar ‘Desiree’ at 0–108 hpi. Different lower case letters show significant differences at *p*< 0.05. Error bars represent the standard deviation (SD) of three biological replicates.

The relative expression levels of *StRab5b* in ‘ZHB’, ‘XPD’, ‘L7’, ‘DXR’, ‘ZS4’, and ‘DXY’ showed a decreasing tendency (**Figure 2A**). The expression profiles indicated that the expression levels of *StRab5b* differed substantially among different organs; the highest expression was in young leaves, followed by expression in stems, old leaves, and roots (**Figure 2B**). The tendency of symptoms in ‘Desiree’ leaves at different time points increased firstly at 12 hpi, followed by reaching the highest point 72 hpi, then dropping to basal level at 108 hpi, indicating the induction of *StRab5b* by *P. infestans* (**Figures 2C, D**).

### 3.3 Transient expression enhanced resistance and gene silencing reduced resistance to *P. infestans* in *N. benthamiana* and potato

**Figure 3:**
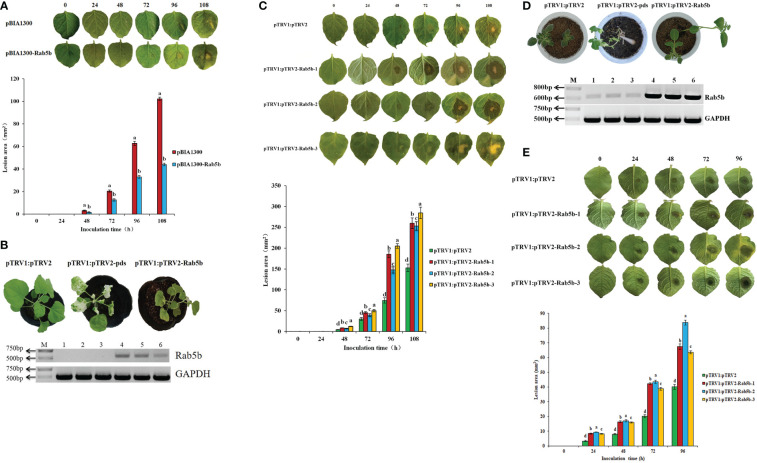
The function of the *StRab5b* gene was verified by both transient expression and VIGS in *Nicotiana benthamiana* and potato **(A)** The differences of lesion in *Nicotiana benthamiana* inoculated after transient expression of *StRab5b* homologous gene at 0–108 hpi. **(B)** The Establishment of VIGS system and identification of *StRab5b* homologous gene silencing efficiency in *N. benthamiana*. Results were obtained from three biological replicates. Photobleaching occurs after 25 dpi. Lanes 1-3 represents the plants injected with pTRV1:pTRV2-*Rab5b*, and lanes 4-6 represents the plants injected with pTRV1:pTRV2 **(C)**The leaf symptoms and the lesion area size of silenced *StRab5b* homologous gene in *Nicotiana benthamiana* at different times. **(D)** The formation of VIGS system in potato and determination of *StRab5b* gene silencing efficiency. Results were obtained from three biological replicates. Photobleaching occurs after 60 dpi. Lanes 1-3 represents the plants injected with pTRV1:pTRV2-*Rab5b*, and lanes 4-6 represents the plants injected with pTRV1:pTRV2. **(E)**The leaf symptoms and the lesion area size of silenced *StRab5b* gene in potato at different times. Error bars represent the standard deviation (SD) of three biological replicates. Different lower case letters indicate significant differences at *p*< 0.05.

Transient expression of *StRab5b* in *N. benthamiana* leaves was performed *via* infiltration, followed by inoculation with *P. infestans*. The lesions on the control leaves gradually increased after inoculation, and reached the largest size (102.1 mm^2^) at 108 hpi; it is much bigger than the lesion size on leave which transiently expressed *StRab5b*, indicating that transiently ectopic expression of *StRab5b* could enhance *N. benthamiana* resistance to *P. infestans* (**Figure 3A**).

To verify the function of *StRab5b* on regulating the resistance to *P. infestans*, VIGS was used to silence *StRab5b* and *NbRab5b* in potato and *N. benthamiana* respectively. *PDS* gene was taken alone as an indicator. When photobleaching was observed on *N. benthamiana* and potato leaves at 25 and 60 dpi, respectively, the expression of *StRab5b*, *NbRab5b* and *PDS* genes was quantified using qRT-PCR and the average silencing efficiencies of *StRab5b*, *NbRab5b* and *PDS* were 59%, 65%, and 100%, respectively (**Figures 3B, D**). The leaf lesion was recorded upon inoculation with *P. infestans*. The lesion area on the infiltrated leaves was 259.9, 253.4, 284, and 152.8 mm^2^ at different time points, which were significantly larger than that on control leaves at the same time point. The average increase in the rate of the lesion area was 3.1 mm^2^/h (**Figure 3C**). However, the leaf lesion areas of potato were 67.4, 83.7, 63.6, and 40.1 mm^2^ at 96 hpi, respectively (**Figure 3E**).

### 3.4 Overexpression of *StRab5b* enhanced potato resistance to *P. infestans*

**Figure 4:**
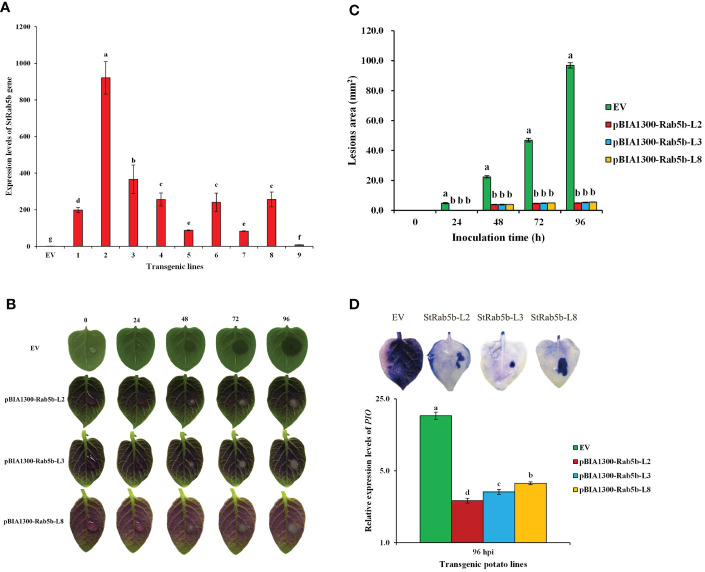
Identification of resistance to *Phytophthora infestans* in transgenic potato plants **(A)** The relative expression levels of *StRab5b* gene in 9 transgenic lines. Different lower case letters indicate significant differences at *p*< 0.05. Error bars represent the standard deviation (SD) of three biological replicates. **(B)** The differences of lesion area on transgenic plants inoculated with *P. infestans* at 0−96 h Results were obtained from five biological replicates. **(C)** Quantification of the lesion area on transgenic plants inoculated with *P. infestans* at 0−96 h Different lower case letters indicate significant differences at *p*< 0.05. Error bars represent the standard deviation (SD). **(D)** Trypan blue staining and the amount of colonization after inoculated with *P. infestans* in transgenic leaves. Results were obtained from three biological replicates. *PIO* gene is an indicator of *P. infestans* and *Actin* is a reference gene.

Positive transgenic lines were obtained *via* Agrobacteria-mediated transformation of *StRab5b* in potato cultivar ‘Desiree’ (**Figure 4A**). The relative expression levels of *StRab5b* among the nine transgenic lines were variable, but significantly higher than its expression levels in control plants. After 96 hpi, the leaf lesions among three transgenic lines (2, 3, and 8) did not differ extensively (5.1, 5.4, and 5.6 mm^2^, respectively) (**Figure 4C**). Meanwhile, the lesions on control leaves increased gradually and reached 96.9 mm^2^ at the same inoculation point (**Figure 4B**). The dead cells on potato leaves were monitored with trypan blue staining; much less blue color was observed on transgenic leaves, indicating a lower number of dead cells on transgenic leaves compared with those in control leaves (**Figure 4D**). However, Quantitative analyze the amount of colonization of *P. infestans* showed that there were significant differences among the three transgenic lines and EV. In addition, the phenotypic characteristics of transgenic plants showed that the stems, leaves and tubers of transgenic plants presented as red, while the control remained green (**Supplementary Figures 1A, B**). The anthocyanin accumulation in leaves and tubers of transgenic lines were greatly higher than that in the EV control (**Supplementary Figure 1C**).

### 3.5 H_2_O_2_ is involved in StRab5b-mediated resistance to *P. infestans*

**Figure 5:**
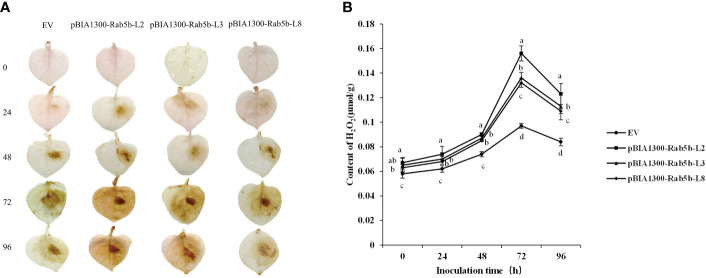
Qualitative and quantitative detection of H_2_O_2_ accumulation after inoculation with *Phytophthora infestans* **(A)** DAB staining to detect H_2_O_2_ accumulation on transgenic plants. Leaves of transgenic plants were inoculated with *Phytophthora infestans* on the right side, while water was used on the left side as control treatment. Three biological replicates were prepared. Lesion expansion was observed at 0, 24, 48, 72, and 96 hpi. **(B)** Accumulation of H_2_O_2_ on transgenic plants. Different lower case letters indicate significant differences at *p*< 0.05. Error bars represent the standard deviation (SD) of three biological replicates.

DAB staining was performed to detect whether H_2_O_2_ was involved in the resistance of *StRab5b* to late blight. A brownish color was observed in the *StRab5b* transgenic leaves at 24 hpi and the color deepened at 48 and 96 hpi. However, no signal was observed in control leaves at 24 hpi, and brownish color on control leaves was lighter than that on the transgenic leaves (**Figure 5A**). The quantification of H_2_O_2_ accumulation is shown in **Figure 5B**. The accumulation of H_2_O_2_ increased after inoculation, peaked at 72 hpi, then decreased in three transgenic and control plants. Compared with the H_2_O_2_ accumulation level in control (0.097 μmol/g), the highest H_2_O_2_ level of 0.156 μmol/g was detected in transgenic line 2 at 72 hpi, followed by other two transgenic lines 3 and 8 with values of 0.136 and 0.132 μmol/g, respectively. The value of H_2_O_2_ was significantly higher than that in control plants after inoculation. These results suggested that *StRab5b* positively regulates potato resistance through H_2_O_2_ accumulation.

### 3.6 The activities and related-genes transcripts of antioxidant enzymes increased after inoculation

**Figure 6:**
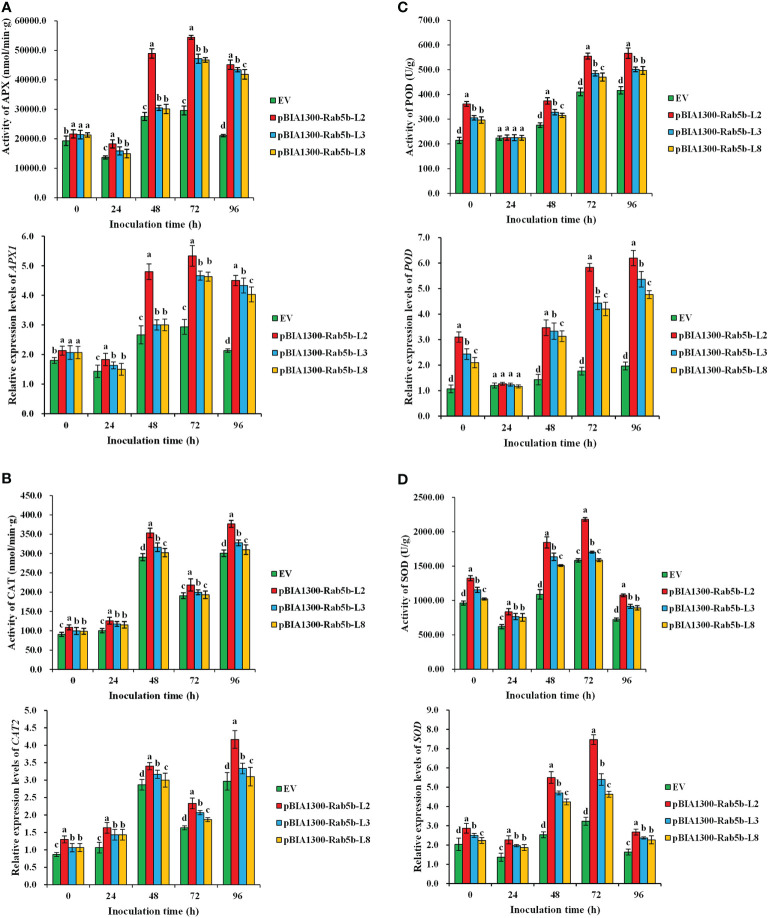
Antioxidant enzyme activities and relative expression levels of genes in transgenic plants after inoculation with *Phytophthora infestans* **(A)** The differences of Ascorbate peroxidase activity and expression levels of *APX1* in transgenic plants at 0−96 hpi. **(B)** The differences of Catalase activity and expression levels of *CAT2* in transgenic plants at 0−96 hpi. activity (0−96 h). **(C)** The differences of Peroxidase activity and expression levels of *POD* in transgenic plants at 0−96 hpi. **(D)** The differences of Superoxide dismutase activity and expression levels of *SOD* in transgenic plants at 0−96 hpi. Different lower case letters indicate significant differences at *p*< 0.05. Error bars represent the standard deviation (SD) of three biological replicates.

Four antioxidant enzyme activities (APX, CAT, POD, and SOD) were monitored after inoculation. The results suggested that APX, CAT, POD, and SOD activities in *StRab5b*-transgenic leaves were substantially higher than those in control plants at different inoculation time points (**Figure 6**). Particularly, SOD, POD, and APX showed a decreasing to- increasing pattern after inoculation, whereas, CAT showed an increasing to- decreasing pattern. Maximum activities of APX and SOD appeared at 72 hpi (54408.5 nmol/min·g and 2180.2 U/g), maximum activities of POD were 566.8 U/g at 96 hpi, except CAT, which had 2 peaks at 48 hpi and 96 hpi (353.1 and 376.8 nmol/min·g), respectively. The expression levels of these four genes (*APX1*, *CAT2*, *POD*, and *SOD*) that encode ROS scavenge enzymes were examined using qPCR after inoculation. The relative expression levels of four genes are consistent with the trend of enzyme activities. The maximum relative expression levels of *APX1*, *CAT2*, *POD*, and *SOD* genes were 5.3, 4.2, 6.2, and 7.5-fold, respectively.

### 3.7 JA is involved in *StRab5b*-mediated potato resistance to *P. infestans*

**Figure 7:**
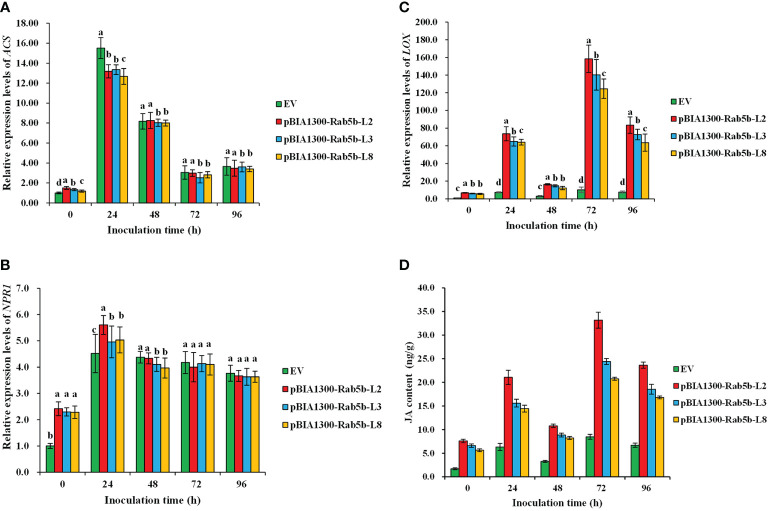
Transcriptional levels of jasmonic acid JA-, salicylic acid SA- and ethylene ET-related genes in transgenic plants after inoculation with *Phytophthora infestans*. **(A)** Relative expression levels of *ACS*. *ACS* gene expression levels were examined by qPCR at 0–96 hpi. **(B)** Relative expression levels of *NPR1*. *NPR1* gene expression levels were examined by qPCR at 0–96 hpi. **(C)** Relative expression levels of *LOX*. *LOX* gene expression levels were examined by qPCR at 0–96 hpi. **(D)** Determination of JA content in transgenic potato lines inoculated with late blight. The JA content was examined using ELISA at 0–96 hpi. Different lowercase letters show significant differences at *p*< 0.05. Error bars represent the standard deviation (SD) of three biological replicates.

Signaling pathways associated with SA, JA, and ET are directly involved in plant resistance to pathogens. To determine whether JA, SA, or ET are involved in *StRab5b*-mediated resistance to *P. infestans*, several marker genes related to different signaling pathways were analyzed at different time points. The results suggested that the expression levels of *LOX* and JA content in three transgenic lines (2, 3, and 8) were induced at 24 hpi and subsequently dropped at 48 hpi, followed by reaching the highest level at 72 hpi (158.2, 140.2, and 124.5-fold expression levels in the *LOX* gene, and 33.2, 24.4, and 20.7 ng/g JA content, respectively); moreover, JA content in transgenic plants was significantly higher than that in EV. Whereas, both *NPR1* and *ACS* showed a different induction pattern compared with the *LOX* gene. They reached the peak at 24 hpi, then decreasing gradually. However, if we compared to the expression of the three marker genes, the induction of *LOX* was much more dramatic than *ACS* or *NPR1*. The induction rate was 67.5-fold at 24 hpi and 141.0-fold at 72 hpi separately, indicating that JA was the dominant molecular signal, which was involved in *StRab5b*-mediated resistance against *P. infestans* (**Figure 7**).

## 4 Discussion

### 4.1 Analysis of *StRab5b* expression profile and induction

Protein StRab5b is an important member of the small G protein Rab family that differs from previously characterized StRab proteins in amino acid structure and domains. The conserved domain motif RabF2 (YYRGA) of the StRab5b was analyzed using multiple sequence alignment. The guanine-binding motif NKAD and the guanine-binding and dissociation motif ETSA were consistent with the expression of TaRab5b protein (Chen et al., 2005). The specific motifs of G1, G3, G5, RabF4, and RabF5 were consistent with those of *Dunaliella salina* and potato StRab proteins. Although the homology of potato *StRab* and *StRab5b* was only 46% at amino acid level, StRab5b showed a structural and functional similarity to the Rab protein (Gao et al., 2012; Yu et al., 2013). Therefore, we replicated the *StRab5b* gene, analyzed its expression profile, and induced expression.

In this study, the relative expression levels of StRab5b were variable among different tissues. The highest expression level was detected in young leaves, followed by stems, old leaves and roots. This is in line with the order of the relative expression levels of another small G protein, *StRac* (Zhang et al., 2020). Additionally, the relative expression levels of *StRab5b* among different varieties is also variable, the order from higher to lower is ZHB, DXR, L7, ZS4, DXY, and XPD. It is also partially confirmed by the results that the relative expression of *StBAG3* was also highest in ‘Zihuabai’ (Lu et al., 2017). Such different expression profiles are likely genotype-dependent. The clear induction pattern of *StRab5b* was also confirmed in ‘Desiree’, indicating the response of *StRab5b* to *P. infestans* infection.

### 4.2 Silencing of *Rab5b* gene negatively regulates resistance to *P. infestans*

Virus-induced gene silencing (VIGS) is an important method for studying gene function and has been used routinely in *N. benthamiana, Lycopersicum esculentum*, and *A. thaliana* (Ratcliff et al., 2001; Brigneti et al., 2004; Lacomme and Chapman, 2008; Senthil-Kumar and Mysore, 2014). In the present study, the *StRab5b* silenced leaves were more susceptible to *P. infestans* than control leaves (**Figure 3**). Similar studies have been reported in *N. benthamiana* and potato. Specifically, small GTPase *NbRanBP1-1*-silencing reduced resistance to *P. infestans* in plants of *N. benthamiana* (Mizuno et al., 2019). Consistently, when silencing *EIN2*, which was involved in pre-invasion phase, facilitated the penetration rate of *P. infestans* (Rin et al., 2017). However, the silencing of *Matrix metalloprotease 1* made *N. benthamiana* more susceptible to *P. infestans*, but enhanced Nep1-like protein induced cell death (Ha et al., 2017). Silencing five candidate genes *TRV:12* (*sodium dicarboxylate cotransporter*), *TRV:17* (*UDP-arabinose 4-epimerase*), *TRV:21* (*2-oxoglutarate dehydrogenase*), *TRV:22* (Lipoxygenase), and *TRV:48* (*suberization-associated anionic peroxidase*) from *N. benthamiana* facilitated the infection of *P. infestans* (Du et al., 2013). Conversely, silencing *StERF3* in potatoes not only enhanced foliage resistance to *P. infestans*, but also promoted plant tolerance to salt stress (Tian et al., 2015).

### 4.3 Overexpressing *StRab5b* in transgenic lines regulates the resistance to *P. infestans* by manipulating H_2_O_2_ accumulation

Currently, small G proteins reportedly respond to abiotic stress factors and regulate plant defense or stress-related gene expression (Miao et al., 2018). The H_2_O_2_ was previously recognized as a toxic metabolite in plant cells. However, it has been known that H_2_O_2_ also mediates stimulus responses to plant cells as a signaling factor (Bailey-Serres and Mittler, 2006). According to previous research, small G proteins of the RAB family play important roles in the establishment of plant defense responses (Lamb and Dixon, 1997). In this study, the results confirmed that H_2_O_2_ accumulation in the transgenic lines might be caused by *StRab5b* expression on the membrane. Previous studies on rice disease resistance have shown that small GTPase *OsRac1* induces hydrogen peroxide products, and enhances resistance to rice blast disease in transgenic plants (Ono et al., 2001). Overexpression of *NtRop* activated H_2_O_2_ products in Arabidopsis transgenic lines (Cao et al., 2008). In potato, stable expression *DN-AtRop1* led to H_2_O_2_ accumulation associated smaller lesions on leaves after inoculation with *P. infestans* (Zhang et al., 2014). Among Rab proteins, overexpression of *StRab* in potato reduced the leaf lesion area and increased H_2_O_2_ production after inoculation with *P. infestans* (Gao et al., 2012). However, less H_2_O_2_ was observed in miR172a transgenic tomato plants after inoculation with *P. infestans* than in the wild type (Luan et al., 2018). These findings indicate that the involvement of H_2_O_2_ in disease resistance is a complex phenomenon, and that H_2_O_2_ accumulation in plant tissues experiences early and late stages. Furthermore, when plants lose the ability for regulation in the late stage, H_2_O_2_ may be induced in the early or late stages after inoculation, and may thus increase in concentration, leading to the cell death.

### 4.4 Antioxidant enzyme activities and key genes are activated in *StRab5b* transgenic lines

In plants, H_2_O_2_ is mainly produced through NADPH oxidase and polyamine oxidase (PAO) pathways. The production of H_2_O_2_ in plants generally suffer abiotic stress through the POD pathway. The extracellular PH value temporarily increases, which can activate the peroxidase of cell wall, and reduces O_2_ to H_2_O_2_ (Khokon et al., 2010). The activities of SOD and POD were higher in miR172 transgenic tomato plants after inoculation with *P. infestans*, accompanied by less H_2_O_2_ (Luan et al., 2018). As shown in **Figure 6**, the inoculated *StRab5b*-transgenic plants had higher APX, SOD, POD, CAT activities and expression levels of ROS related-genes, and increased H_2_O_2_ accumulation. These results suggested that *StRab5b* in potato may modulate antioxidants to eliminate redundant H_2_O_2_ and prevent cellular membrane injury after *P. infestans* infection. Similar results have been found in tomato, and the amounts of H_2_O_2_ and O_2_^−^ were increased with the continuous salt stress in both roots and leaves. Compared to control, the plants had increased activities of SOD, CAT, POD, and APX after 6 or 12 days of salt stress (Raziq et al., 2022). In tomato, the melatonin-induced H_2_O_2_ generation through RBOH was attributed to activate some genes expression (*CDPK1*, *MAPK1* and *ERD15*). Conversely, the inhibition of RBOH reduced stress defense response and antioxidant enzyme activity (SOD, CAT and APX) (Gong et al., 2017). In addition, a previous study reported that *APX1* and *SOD* genes showed higher levels in SA- or CdCl_2_-treated potato plants, when compared to CK plants (Li et al., 2019). Brassinosteroid (BR) activated the expression of the *StPOD* gene, and the expression level was partially increased in potato tubers at the end of wound healing (Han et al., 2022). Some researchers believe that the highly active antioxidant enzymes are used to eliminate active oxygen, and the concentration of H_2_O_2_ is reduced.

### 4.5 Potato JA-mediated resistance to late blight is regulated by *StRab5b*

Plant defense responses refer to the resistance of plants to external infection, including the synthesis of pathogenesis-related (PR) proteins. Plant hormones SA, JA, and ET play major roles in the plant immune system (Verma et al., 2016). There are many studies regarding plant resistance and JA signaling pathway. For example, JA may be involved in resistance regulated by AtRop1 (Zhang et al., 2014). Overexpression of *OsRab11* in *Arabidopsis* enhances plant resistance to *Pseudomonas syringae* by inducing the expression of JA-responsive genes, rather than SA-responsive genes (Hong et al., 2013). These results are consistent with those obtained in the present study. Thus, the key enzyme-encoding gene in the JA signaling pathway, i.e., *LOX* and JA content were dramatically induced in *StRab5b* transgenic lines at different inoculation time points, indicating the involvement of JA signaling pathway on potato resistance manipulated by *StRab5b.* This is in contrast with the enhanced late blight resistance observed by the overexpression of LecRK-I.9 in *Arabidopsis* by reducing SA-responsive gene expression (Bouwmeester et al., 2014). SA and ROS were confirmed as the primary signaling molecules that mediated potato resistance to *P. infestans* (Lamb and Dixon, 1997; Na et al., 2012), and the present study clearly highlights the involvement of the JA pathway in potato resistance to *P. infestans*. However, the cross talk of JA and SA signaling pathways were confirmed *via* hormone-responsive transcription factors WRKY70 and PDF1.2, which regulate plant defense responses (Petersen et al., 2000; Li et al., 2004). In other words, the mechanism underlying the involvement of small G proteins on manipulating potato resistance against late blight resistance is rather complex, and different signaling pathways interact with each other through key transcription factors to improve durable resistance in solanaceous crops. Whether the same mechanism occurs in small G protein-mediated establishment of potato resistance remains to be explored. Further experiments are needed to delineate the signaling transduction pathway mediated by small G proteins in potato.

## Data availability statement

The original contributions presented in the study are included in the article/**Supplementary Material**, further inquiries can be directed to the corresponding author/s.

## Author contributions

ZT and ZZ conceived the experiments. JuZ designed and guided the experiments scheme. ZT, ZZ, LK and ML completed the experiments. ZT, XG, JY, JiZ and YF provided consumable materials/reagents/software and analyzed the data. ZT wrote the paper. All authors contributed to the article and approved the submitted version.
