# Supplementary material for: Small G Protein StRab5b positively regulates potato resistance to Phytophthora infestans
Source: Front Plant Sci. 2023 Jan 9;13:1065627. doi: 10.3389/fpls.2022.1065627 (PMC9868449; doi:10.3389/fpls.2022.1065627)

## Supplementary Material

# Supplementary Table 1 Primer sequences designed for this study

| Primers name | Base sequences (5ʹ to 3ʹ) |
| --- | --- |
| StRab5b-F | GAAGATCTTCATGGGTTGCGCATCTTCAGC (*Bgl*II) |
| StRab5b-R | GGGGTACCCCATGATCAAGCAGCAGTCG (*Kpn*I) |
| StGAPDH-F | TGGACAATGGAAGCACCATGAGC |
| StGAPDH-R | TGCTTGACCTGCTGTCACCAAGA |
| PIO8-3-3F | CAATTCGCCACCTTCTTCGA |
| PIO8-3-3R | GCCTTCCTGCCCTCAAGAAC |
| VIGSRab5b-F | TGCTCTAGACTTAATCCTGATAATGGTGG (*Xba*I) |
| VIGSRab5b-R | CGGGGTACCCTGGTTGATATTATCAGCTGTC (*Kpn*I) |
| VIGSPDS-F | GCTCTAGAGCGGGTGGTTTGTCTACAGC |
| VIGSPDS-R | GGGGTACCCCACACTCCCATCCTCATTCAACTCAA |
| StActin-F | GATGGTGTCAGCCACAC |
| StActin-R | ATTCCAGCAGCTTCCATTCC |
| poACS-F | TCCTGGTGATGCATTTCTAGTTCCT |
| poACS-R | ATCCATCCAAATAAATAGGCCAGCAT |
| LOX-F | CTTATGTTGCGGTGAATGACGTTGG |
| LOX-R | CCTGCATAAGGATATTGCCCGAAA |
| NPR1-2F | TTACCAAGTCTACAGAGGAAGGAA |
| NPR1-2R | AATCATCGCCTGCCATAGC |
| NbGAPDH-F | CAGGAACCCTGAAGATATCCC |
| NbGAPDH-R | GCAGTTGGTACTCTGAAGGCC |
| M13-R | CAGGAAACAGCTATGACC |
| M13-F | TGTAAAACGACGGCCAGT |
| q-StRab5b -F | ATAGCCTTGCAGGACTCAAC |
| q-StRab5b -R | CAGCACCTCGGTAGTATAATGG |
| StSOD -F | CCGACAAGCAGATTCCTCTC |
| StSOD -R | CAGGAGCAATTAACCCTGGA |
| StAPX1 -F | GCCTTCTTCGCTGACTATGC |
| StAPX1 -R | TCCAGCGAGCTTTTCAGAAT |
| StPOD -F | AGGGACTGCTCCATTCTG |
| StPOD -R | CGGTTATCACCCATCTTA |
| StCAT2 -F | GTGGTCAGAAGGTTGCTTCTCGTCT |
| StCAT2 -R | GATGATGTTCTTCAAGCACCAAAGTG |

## Supplementary Figure 1 Phenotypic identification of StRab5b transgenic plants

## (A) The differences of leaf colors in transgenic plants that were cultured for 55 d. (B) Phenotypic characteristics of potato tubers from transgenic plants. (C) Determination of anthocyanin content in leaves and tubers of transgenic plants. Different lower case letters show significant differences at *p* ＜ 0.05. Three samples of both potato leaves and tubers were collected for data measurements.


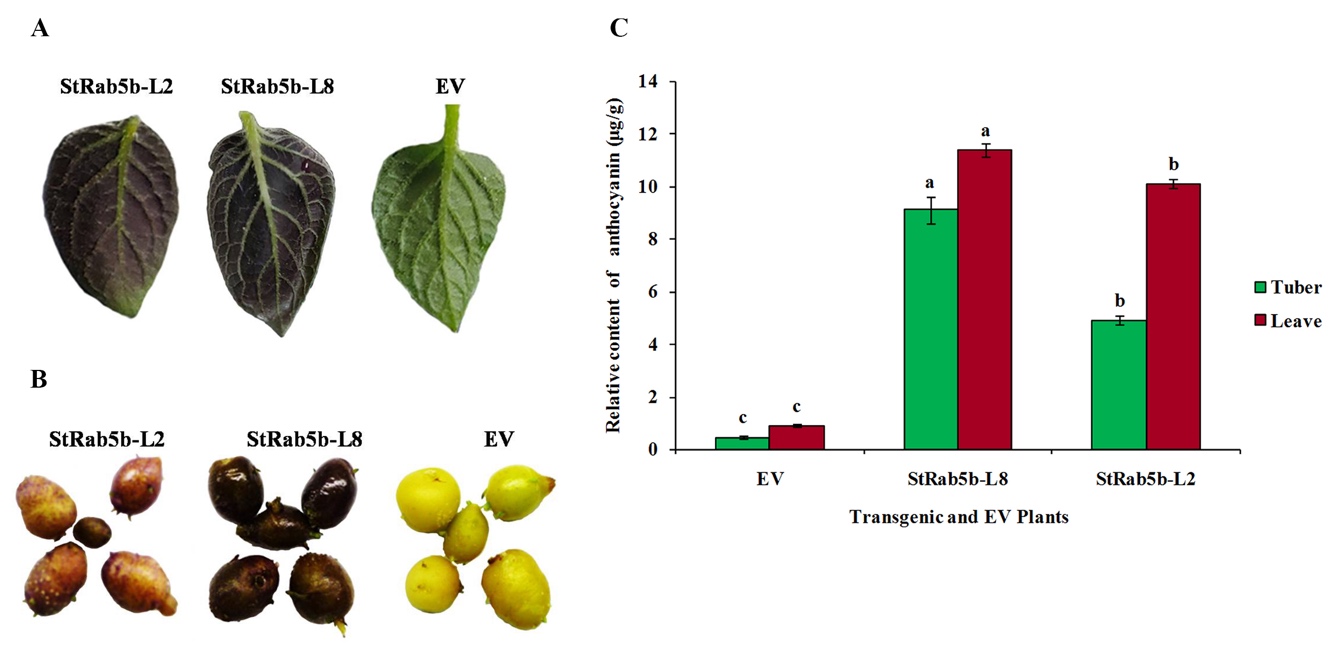

Supplement: Supplementary file 1 [file DataSheet_1.docx]
